# Reconfigurable Sensor Analog Front-End Using Low-Noise Chopper-Stabilized Delta-Sigma Capacitance-to-Digital Converter

**DOI:** 10.3390/mi9070347

**Published:** 2018-07-10

**Authors:** Hyungseup Kim, Byeoncheol Lee, Yeongjin Mun, Jaesung Kim, Kwonsang Han, Youngtaek Roh, Dongkyu Song, Seounghoon Huh, Hyoungho Ko

**Affiliations:** 1 Department of Electronics Engineering, Chungnam National University, Daejeon 34134, Korea; hyungseup@cnu.ac.kr (H.K.); dada5891@cnu.ac.kr (B.L.); ansdud159@cnu.ac.kr (Y.M.); jskim1@cnu.ac.kr (J.K.); kshan1@cnu.ac.kr (K.H.); 2 LeoLSI Co., Ltd., Seoul 06728, Korea; ytroh@leolsi.com (Y.R.); dksong@leolsi.com (D.S.); iamtoto@leolsi.com (S.H.)

**Keywords:** capacitive microsensor, analog front-end (AFE), capacitive sensor interface circuit, reconfigurable sensor readout circuit, delta-sigma modulation, capacitance-to-digital converter (CDC), temperature sensor, low-noise technique, chopper stabilization

## Abstract

This paper proposes a reconfigurable sensor analog front-end using low-noise chopper-stabilized delta-sigma capacitance-to-digital converter (CDC) for capacitive microsensors. The proposed reconfigurable sensor analog front-end can drive both capacitive microsensors and voltage signals by direct conversion without a front-end amplifier. The reconfigurable scheme of the front-end can be implemented in various multi-mode applications, where it is equipped with a fully integrated temperature sensor. A chopper stabilization technique is implemented here to achieve a low-noise characteristic by reducing unexpected low-frequency noises such as offsets and flicker noise. The prototype chip of the proposed sensor analog front-end is fabricated by a standard 0.18-μm 1-poly-6-metal (1P6M) complementary metal-oxide-semiconductor (CMOS) process. It occupies a total active area of 5.37 mm^2^ and achieves an effective resolution of 16.3-bit. The total power consumption is 0.843 mW with a 1.8 V power supply.

## 1. Introduction

The emergence of the Internet of Things (IoT) in recent years has greatly influenced the field of electronics, and the demand for the development of IoT based applications has increased. In particular, as the demand for various IoT applications grows, the performances of the sensor and the sensor interface integrated circuits have become increasingly important. Low-noise characteristics and low power consumption of the sensor interface circuits have become essential requirements. Capacitive microsensors are widely adopted in various applications such as humidity sensors, accelerometers, gyroscopes, biological sensors, pressure sensors, touch screen sensors, and proximity sensors [1,2,3,4,5,6,7,8,9]. With the wide use of capacitive microsensors, many research works on capacitive microsensor interface circuit techniques have been reported [10,11,12,13,14,15,16,17,18,19,20,21,22]. A multi-stage amplification capacitive sensor readout circuit with parasitic capacitance cancellation technique has been presented [10,11]. However, the multi-stage amplification scheme requires high power consumption and a large active area, which are not suitable for IoT applications that demand low power consumption and small size. A successive approximation register (SAR) capacitance-to-digital converter (CDC) scheme for low-power applications has been presented [12,13,14,15,16]. The SAR CDC scheme can achieve low power consumption and small size; however, it severely suffers from the effect of parasitic capacitance due to the direct connection of the capacitive sensor to the input of the comparator without the pre-amplifier. The implementation of the pre-amplifier in the SAR CDC scheme can relieve the effect of the parasitic capacitance, but the circuit complexity and power consumption increase. A delta-sigma modulation is widely used for high resolution capacitance-to-digital conversion. A capacitive sensor readout circuit with charge sensing amplifier (CSA) for pre-amplification of the sensor signal and a delta-sigma modulator can achieve high resolution because of its low-noise characteristic [17], however, the chip size and power consumption increase due to the additional CSA. To reduce the area and power consumption, the direct conversion delta-sigma CDC can be a good solution. The direct conversion first-order delta-sigma CDC scheme can relieve the problems by directly converting the capacitance change of the capacitive sensor to digital codes [18,19,20,21]. However, the first-order delta-sigma modulation scheme suffers from the dead zone problem, which increases the nonlinearity error and leads to performance degradation [22,23,24,25].

This paper presents a reconfigurable sensor analog front-end using low-noise chopper-stabilized delta-sigma CDC. The main strength of the proposed sensor analog front-end is the reconfigurable scheme, which can drive capacitive sensors and voltage signals without a front-end amplifier by direct conversion in the second-order incremental delta-sigma converter scheme. It also employs a chopper stabilization technique to achieve a low-noise characteristic. The second-order delta-sigma scheme can relieve the dead zone problem of the first-order delta-sigma modulation scheme, which causes nonlinearity error and performance degradation. The proposed sensor analog front-end with fully integrated temperature sensor can be flexibly adopted in various IoT sensor system applications due to its reconfigurable scheme.

This paper is organized as follows: Section 2 discusses the circuit implementation of the proposed reconfigurable sensor analog front-end. Section 3 describes the measurement results of the proposed reconfigurable sensor analog front-end. Finally, Section 4 presents the discussion of the proposed reconfigurable sensor analog front-end by comparison of performance with previously reported works and presents the conclusions of this work.

## 2. Circuit Implementation

### 2.1. Top Level Architecture

**Figure 1:**
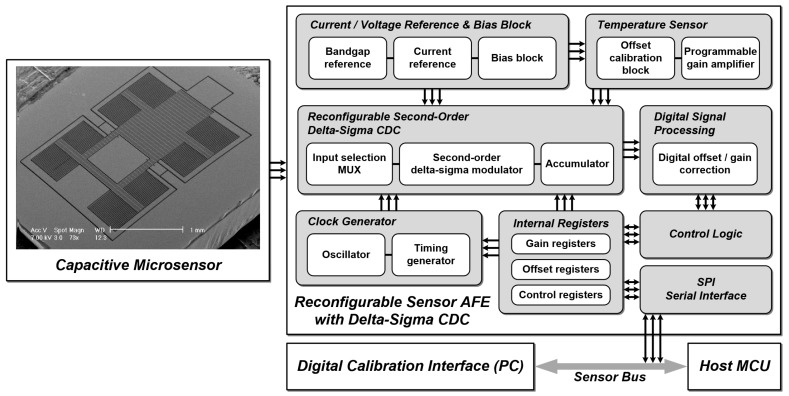
Block diagram of the proposed reconfigurable sensor analog front-end.

The block diagram of the proposed reconfigurable sensor analog front-end is shown in Figure 1. The analog front-end circuit includes: a second-order incremental delta-sigma CDC; a current/voltage reference block with the bandgap reference and bias block with a resister string; a clock generator with an on-chip oscillator and timing generator; a fully integrated temperature sensor; a digital offset/gain correction block; and a serial peripheral interface (SPI) for interface with the host microcontroller unit (MCU). The basic scheme of the reconfigurable sensor analog front-end comprises a second-order delta-sigma modulation scheme, which relieves the nonlinearity error and performance degradation caused by the dead zone that appears when the first-order delta-sigma modulation scheme is adopted. The reconfigurable sensor analog front-end can drive capacitive microsensors and voltage signals without a front-end amplifier by direct conversion. The proposed scheme can drive the capacitive microsensors in single drive mode or differential drive mode depending on the application. The bias generation block contains a bandgap reference, current reference and a bias block. The bandgap reference generates a reference voltage and the current reference generates the reference current with the bandgap voltage. The bias voltages generated in the bias block with the reference current supplies bias voltages for each sub-block. The bandgap reference also generates the complementary to the absolute temperature (CTAT) characteristic voltage for the operation of the fully integrated temperature sensor, which can be incorporated in specific applications to measure temperature when needed. The driving mode of the capacitive microsensors, the voltage signal driving mode, and the on-chip temperature sensor as the voltage signal driving mode, can be selected by register selection. The timing generator generates the clock signals needed by the on-chip oscillator, which can generate 1 MHz, 2 MHz, and 4 MHz master clocks by register selection or by the external input clock signal. The digital offset/gain correction block is integrated for offset/gain calibration. The SPI is used for register control and digital offset/gain correction block control with a laptop computer.

### 2.2. Reconfigurable Sensor Analog Front-End

**Figure 2:**
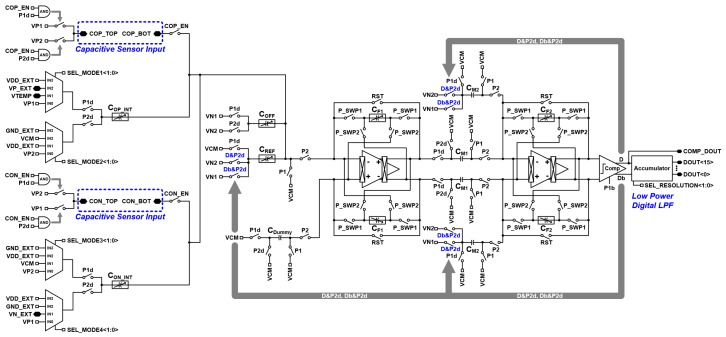
Schematic of the second-order delta-sigma CDC.

**Figure 3:**
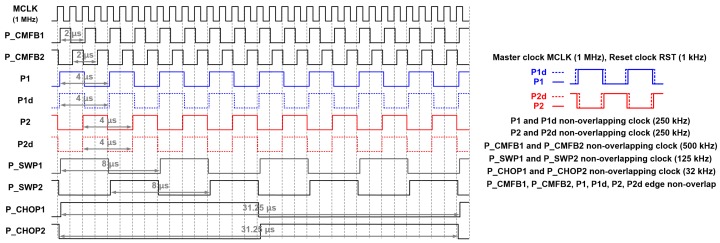
Timing diagram of the second-order delta-sigma CDC.

The schematic of the proposed reconfigurable second-order delta-sigma CDC is shown in Figure 2. The proposed reconfigurable sensor analog front-end is based on the second-order delta-sigma CDC scheme, which operates on the same concept as the conventional delta-sigma modulator based CDC. The switch-capacitor scheme serves to balance the signal charge of the input sensor capacitor (*C_OP_* or *C_ON_*) with the programmable reference capacitor (*C_REF_*) using the feedback capacitor of the integrator (*C_F_*_1_ and *C_F_*_2_), which balances the average charge to zero. The programmable offset capacitor (*C_OFF_*) is used for offset cancellation. The output signal through the two-stage integrator and comparator output is a digital signal, which is converted to 16-bit digital codes by the low-power digital low-pass filter (LPF) based on the accumulator. The digital data of the proposed scheme can also be acquired as bit-stream data (COMP_DOUT) depending on the application preference. The programmable feedback capacitor of the first stage integrator (*C_F_*_1_) and second stage integrator (*C_F_*_2_) can be controlled from 0.177 pF to 22.671 pF by using a 7-bit control register, and from 0.177 pF to 11.336 pF by using a 6-bit control register. The timing diagram of the proposed reconfigurable sensor analog front-end is shown in Figure 3. The on-chip oscillator can generate 1 MHz, 2 MHz, or 4 MHz master clocks by register selection. Moreover, the external input clock can be operated manually. The default master clock (MCLK) operates at 1 MHz. P_CMFB1 and P_CMFB2 are non-overlapping clock signals used in the switch capacitor common mode feedback (SC-CMFB) for the fully differential amplifier, which operates at 500 kHz. *P*1, *P*1*d*, *P*2, and *P*2*d* are non-overlapping clocks operating at 250 kHz. The edges of P_CMFB1, P_CMFB2, *P*1, *P*1*d*, *P*2, and *P*2*d* do not overlap. The feedback capacitor swapping non-overlapping clocks of P_SWP1 and P_SWP2 operate at 125 kHz. The chopper non-overlapping clocks operate at 32 kHz. The reset clock (RST) operates at 1 kHz when implemented with the 1 MHz master clock. The proposed sensor analog front-end adopts a low-noise technique for ensuring a low-noise characteristic.

**Figure 4:**
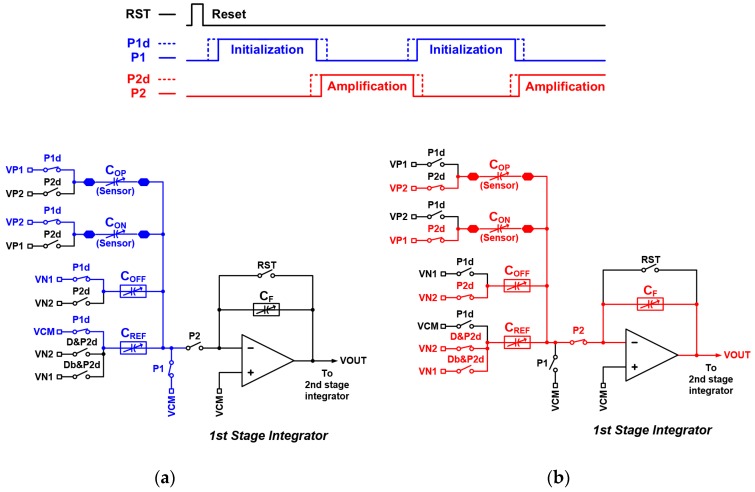
Detailed operation timing of the integrator in the delta-sigma CDC. (**a**) Initialization phase; (**b**) Amplification phase.

The detailed operation timing of the integrator in the delta-sigma CDC is shown in Figure 4 (i.e., the first stage integrator with differential capacitive sensor inputs *C_OP_* and *C_ON_*). The operation is explained with a single-ended circuit scheme for simplicity. The RST is controlled by the accumulator in the back-end. The RST resets both of the first stage and second stage integrators after counting a number of 65,536 comparator output codes in the accumulator. The comparator output signal *D* and inverted signal *Db* are added to *P*2*d* with an AND gate controlling a *VN*1 switch and a *VN*2 switch connected to *C_REF_*. After the reset phase, the CDC operates with two clock phases: An initialization phase and an amplification phase. The initialization phase is shown in Figure 4a. The non-overlapping clocks *P*1 and *P*1*d* enable the input sensors *C_OP_*, *C_ON_* and *C_OFF_* to be charged while *C_REF_* is initialized to be reset by the reference voltage (*VCM*). The total charge can be expressed as Equation (1):

(1) $$Q_{P1\&P1d}=C_{OP}\cdot (VCM-VP1)+C_{ON}\cdot (VCM-VP2)+C_{OFF}\cdot (VCM-VN1)$$

The amplification phase is shown in Figure 4b. The non-overlapping clocks *P*2 and *P*2*d* enable the charges stored in the input sensors *C_OP_*, *C_ON_* and *C_OFF_* to be transferred to the first stage integrator and to be amplified. The total charge in the amplification phase can be expressed as Equation (2):

(2) $$\begin{array}{l} Q_{P2\&P2d}=C_{OP}\cdot (VCM-VP2)+C_{ON}\cdot (VCM-VP1)+C_{OFF}\cdot (VCM-VN2)\\ \;+D\cdot C_{REF}\cdot (VCM-VN2)+Db\cdot C_{REF}\cdot (VCM-VN1)+C_{F}\cdot (VCM-VOUT) \end{array}$$

The total charge during the initialization phase and the amplification phase should be equal. The total charge transferred should satisfy Equation (3):

(3) $$Q_{P1\&P1d}=Q_{P2\&P2d}$$

The voltages *VP*1, *VP*2, *VN*1 and *VN*2 are set according to Equation (4):

(4) VP1=VCM+0.5⋅REFPVP2=VCM−0.5⋅REFPVN1=VCM−0.5⋅REFNVN2=VCM+0.5⋅REFN

By Equations (3) and (4), the simplified output of the integrator can be expressed as Equation (5):

(5) $$\begin{array}{l} VOUT=(\frac{1}{C_{F}})\cdot (C_{OP}\cdot REFP-C_{ON}\cdot REFP-C_{OFF}\cdot REFN\\ \;-0.5\cdot D\cdot C_{REF}\cdot REFN+0.5\cdot Db\cdot C_{REF}\cdot REFN)+VCM \end{array}$$

The output of the first stage integrator is then amplified by the second stage integrator by the same procedure. After the amplification of the second stage integrator, the comparator compares the second stage integrator and outputs bit-stream data.

**Figure 5:**
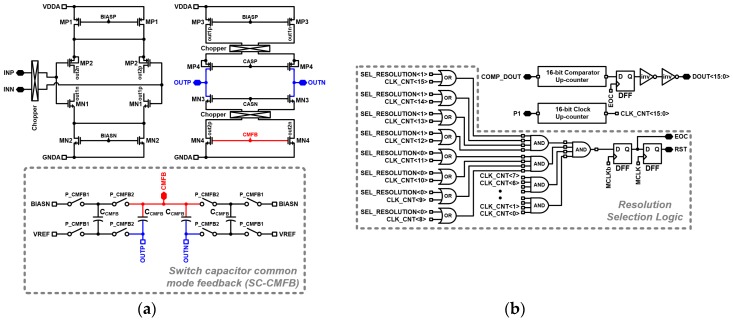
Schematic of fully differential chopper amplifier and accumulator. (**a**) Fully differential chopper amplifier; (**b**) Resolution selectable accumulator.

The chopper stabilization technique is implemented to reduce low-frequency noises such as offsets and flicker noise [26]. The fully differential chopper amplifier is implemented as the amplifier of the integrator to obtain a low-noise characteristic. Similar fully differential chopper amplifiers are implemented for each stage. The schematic of the proposed fully differential chopper amplifier is shown in Figure 5a. The switched-capacitor common mode feedback (CMFB) circuit is implemented for low power consumption which generates the CMFB voltage for the fully differential chopper amplifier. The amplifier is designed with a DC gain of 78.68 dB and a 1.949 MHz unit gain bandwidth (UGBW). A feedback capacitor swapping scheme is adopted to enhance the common mode rejection ratio (CMRR) and thereby reduce the common mode noise of the sensor analog front-end. Figure 5b shows the resolution selectable accumulator. The accumulator consists of up-counters and resolution selection logic. The output digital code resolution can be selected by the accumulator by SEL_RESOULTION<1:0> controlling the resolution selection logic. The resolution of the output digital code can be selected as 8-bit, 12-bit and 16-bit for specific application needs.

The proposed sensor analog front-end can drive both capacitive microsensors and input voltage signals by register selection. The mode selection can be performed by controlling the registers SEL_MODE1<1:0> to SEL_MODE4<1:0> of the analog selection multiplexers, COP_EN and CON_EN. The driving mode of the single capacitive microsensors can be selected by enabling either COP_EN or CON_EN. The driving mode of the differential capacitive microsensors can be selected by enabling both COP_EN and CON_EN. When the driving mode of the capacitive microsensors is enabled, the internal programmable capacitors *C_OP_INT_* and *C_ON_INT_* should be switched off for correct conversion. The capacitive sensing mode operation can be expressed as Equation (6). The computations shown in Equation (6) can be denoted as *REFP* and *REFN*.

(6) VP1−VP2=REFPVN2−VN1=REFN

As shown in Equation (5), the differential output voltage of the second-order delta-sigma integrator of the proposed reconfigurable sensor analog front-end can be defined as Equation (7). The term *C_F_* is the feedback capacitor of the second-order delta-sigma integrator.

(7) $$\begin{array}{cc} \Delta V_{O} & =(\frac{1}{C_{F}})\cdot (C_{OP}\cdot REFP-C_{ON}\cdot REFP-C_{OFF}\cdot REFN-0.5\cdot D\cdot C_{REF}\cdot REFN\\ & +0.5\cdot Db\cdot C_{REF}\cdot REFN) \end{array}$$

The input capacitance range can be defined by each conditions. When the differential output voltage of the second-order delta-sigma integrator is higher than 0 V and the bit-stream data *D*[*n*] is Low (0), it is saturated and the minimum input range can be expressed as Equation (8):

(8) $$\Delta V_{O}>0\;\&\;D[n]=0\;(\mathrm{Minimum}\;\mathrm{input}\;\mathrm{capacitance}\;\mathrm{range})\;(C_{OFF}-0.5\cdot C_{REF})\cdot REFN<(C_{OP}-C_{ON})\cdot REFP$$

When the differential output voltage of the second-order delta-sigma integrator is lower than 0 V and the bit-stream data *D*[*n*] is High (1), it is saturated and the maximum input range can be expressed as Equation (9):

(9) $$\Delta V_{O}<0\;\&\;D[n]=1\;(\mathrm{Maximum}\;\mathrm{input}\;\mathrm{capacitance}\;\mathrm{range})\;(C_{OP}-C_{ON})\cdot REFP<(C_{OFF}+0.5\cdot C_{REF})\cdot REFN$$

The capacitive input range of the driving mode of the capacitive microsensors can be defined as Equation (10):

(10) $$(C_{OFF}-0.5\cdot C_{REF})\cdot \frac{REFN}{REFP}<(C_{OP}-C_{ON})<(C_{OFF}+0.5\cdot C_{REF})\cdot \frac{REFN}{REFP}$$

The programmable capacitors *C_REF_* and *C_OFF_* can each be controlled from 0.177 pF to 11.158 pF by a 6-bit control register; their on and off states can also be controlled. The maximum capacitive input range of the proposed reconfigurable sensor analog front-end is 16.738 pF.

The voltage signal driving mode can be enabled by disabling COP_EN and CON_EN for the driving mode of the capacitive microsensors. When the voltage signal driving mode is enabled, the internal programmable capacitor *C_OP_INT_* or *C_ON_INT_* of the sampling capacitor should be switched on. A single-ended voltage mode can be selected by switching on one of the internal programmable capacitors *C_OP_INT_* and *C_ON_INT_*. When the internal programmable capacitor *C_OP_INT_* is selected as the single-input sampling capacitor, the input signal VP_EXT should be selected by controlling SEL_MODE1<1:0>. Further, when the internal programmable capacitor *C_ON_INT_* is selected as the single-input sampling capacitor, the input signal VN_EXT should be selected by controlling SEL_MODE4<1:0>. A differential voltage mode can be selected by switching on both the internal programmable capacitors *C_OP_INT_* and *C_ON_INT_*. When the differential input mode is selected, the internal programmable capacitors *C_OP_INT_* and *C_ON_INT_* should be switched on as differential sampling capacitors for both inputs. In addition, both the input signals VP_EXT and VN_EXT should be selected by controlling the SEL_MODE1<1:0> and SEL_MODE4<1:0> registers. The switching voltage can be controlled by SEL_MODE2<1:0> and SEL_MODE3<1:0>. The voltage signal driving mode with the single-ended voltage mode with VP_EXT input can be expressed as Equation (11) when the condition is as specified in the equation:

(11) $$VP1-VP2=REFP,\;VP1=V_{IN}$$

From Equations (10) and (11), the input range of the single-ended voltage mode can be deduced as Equation (12):

(12) $$(\frac{C_{OFF}-0.5\cdot C_{REF}}{C_{OP\_INT}})\cdot REFN+VP2<V_{IN}<(\frac{C_{OFF}+0.5\cdot C_{REF}}{C_{OP\_INT}})\cdot REFN+VP2\;VP1=VP\_EXT$$

The single-ended voltage mode with VN_EXT input can be expressed as for Equation (12) by changing the parameters *C_OP_INT_* to *C_ON_INT_*, and VP_EXT to VN_EXT. Each of the programmable capacitors *C_REF_*, *C_OFF_*, *C_OP_INT_*, and *C_ON_INT_* can be controlled from 0.177 pF to 11.158 pF by a 6-bit control register, and their on and off states can also be controlled. The maximum voltage mode input range of the proposed reconfigurable sensor analog front-end is 0 V to 1.8 V.

### 2.3. Fully Integrated Temperature Sensor

**Figure 6:**
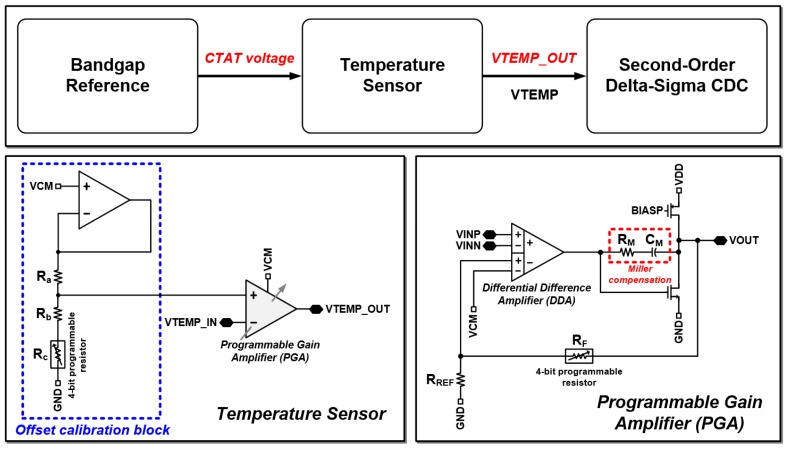
Schematic of fully integrated temperature sensor.

The schematic of the fully integrated temperature sensor of the proposed reconfigurable sensor analog front-end is shown in Figure 6. The temperature sensor operates and senses the temperature with the CTAT voltage generated by the bandgap reference. The output analog voltage of the temperature sensor is converted to digital codes by the voltage input mode of the proposed scheme. The scheme of the temperature sensor has been presented in [27]. The temperature sensor consists of an offset calibration block and the programmable gain amplifier (PGA). The programmable resistor (*R_c_*) of the offset calibration block can be controlled from 52.267 kΩ to 784.016 kΩ. The value of resistor R_a_ is 250.885 kΩ and that of R_b_ is 385.875 kΩ. The offset voltage of the temperature sensor can be expressed as Equation (13):

(13) $$V_{OFFSET}=(\frac{R_{b}+R_{c}}{R_{a}+R_{b}+R_{c}})\cdot VCM$$

The PGA is implemented using a differential difference amplifier (DDA). The gain of the PGA is controlled by the 4-bit programmable resistor *R_F_* from 522.678 kΩ to 7.840 MΩ. The gain of the PGA can be expressed as Equation (14):

(14) $$VOUT=(1+\frac{R_{F}}{R_{REF}})\cdot (VINP-VINN)+VCM$$

Therefore, the output voltage of the temperature sensor can be expressed as Equation (15).

(15) $$VTEMP\_OUT=(1+\frac{R_{F}}{R_{REF}})\cdot (V_{OFFSET}-VTEMP\_IN)+VCM$$

The gain for temperature signal amplification and the offset level can be controlled by the proposed scheme depending on the application status in the temperature range of −10 °C to 120 °C.

**Figure 7:**
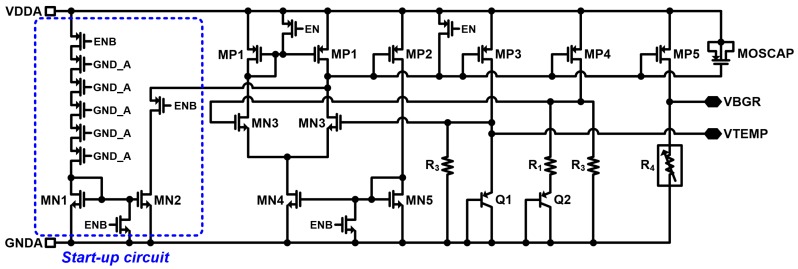
Schematic of bandgap reference of the fully integrated temperature sensor.

The schematic of the bandgap reference of the fully integrated temperature sensor is shown in Figure 7. The implemented low voltage bandgap reference in the temperature sensor is a modified version of the previous bandgap scheme [28]. The bandgap reference operates with the input enable signal (EN) to be High (1) (input disable signal (ENB) to be Low (0)) which operates the start-up circuit. The output bandgap reference voltage (*VBGR*) is generated by ratios of the resistors *R*_1_, *R*_2_, *R*_3_ and *R*_4,_ following Equation (16) (when the ratio between the two bipolar junction transistors (BJT) is *Q*1:*Q*2 = 1:24):

(16) $$VBGR=(\frac{R_{4}}{R_{1}})\cdot V_{T}\cdot ln(n)+(\frac{R_{4}}{R_{3}})\cdot VEB_{Q2}\;ln(24)=3.178$$

**Figure 8:**
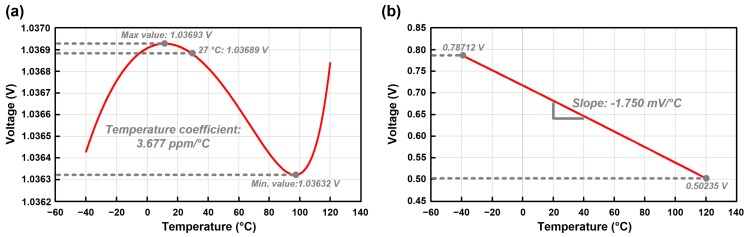
Simulation results of the bandgap reference. (**a**) Temperature sweep of output bandgap reference voltage (VBGR); (**b**) Generated temperature sensor voltage (VTEMP).

The bandgap resistor *R*4 is implemented with a 3-bit programmable resistor for voltage trimming with a range of 90.986 kΩ to 126.280 kΩ. Figure 8 shows the simulation results of the bandgap reference. The simulation result of the temperature sweep in the range of −40 °C to 120 °C is shown in Figure 8a. The simulated temperature coefficient is 3.677 ppm/°C. The generated temperature sensor voltage (VTEMP) with CTAT voltage characteristic is shown in Figure 8b. The simulation result shows that the VTEMP has CTAT characteristic with −1.750 mV/°C, which is used in the proposed on-chip temperature sensor shown as in Equation (15).

## 3. Measurement Results

### 3.1. Prototype Chip Implementation

**Figure 9:**
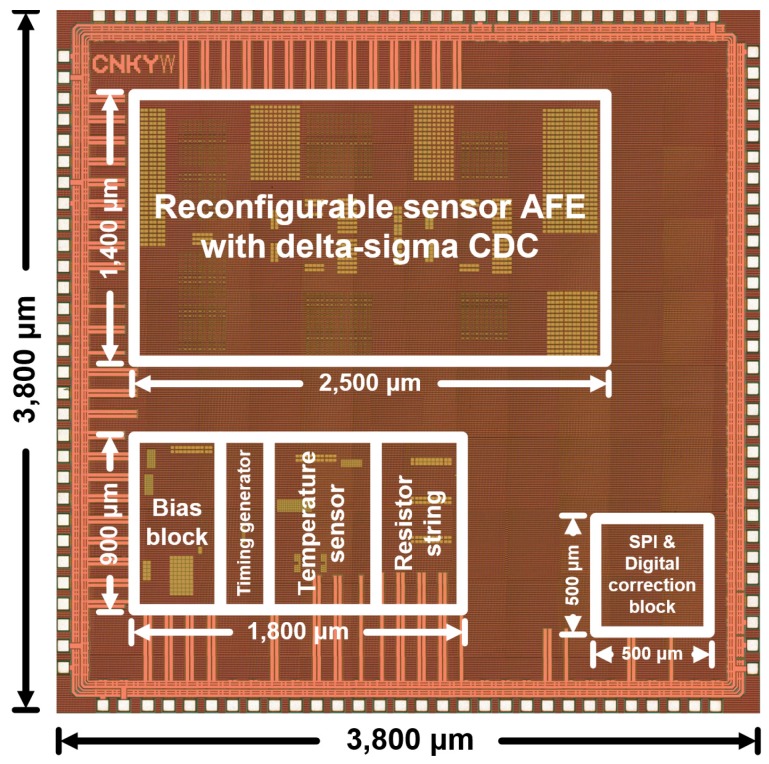
Die photograph of the proposed reconfigurable sensor analog front-end IC.

The reconfigurable sensor analog front-end integrated circuit (IC) was fabricated using a standard 0.18-µm complementary metal-oxide-semiconductor (CMOS) process with an active area of 5.37 mm^2^. The die photograph is shown in Figure 9. The chip is fully integrated without other external elements. The total power consumption is 0.843 mW with 1.8 V power supply.

### 3.2. Measurement Environment

**Figure 10:**
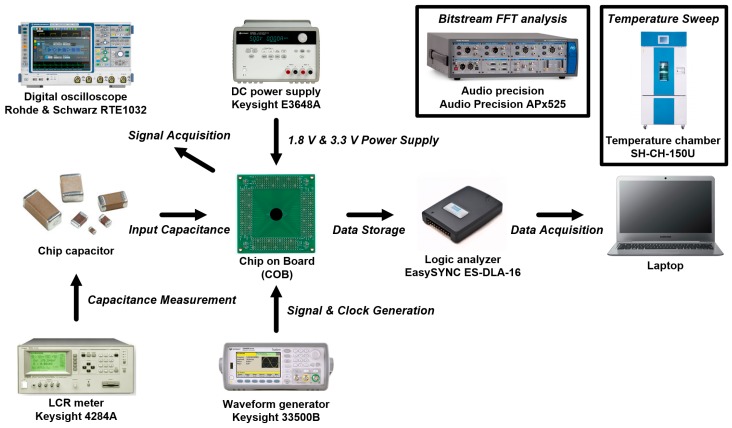
Measurement environment of the proposed reconfigurable sensor analog front-end IC.

The measurement environment of the reconfigurable sensor analog front-end IC is shown in Figure 10. A digital oscilloscope was used for signal acquisition and data analysis. The output digital codes of the prototype IC are acquired by the logic analyzer through the laptop computer. The Fast Fourier Transform (FFT) measurement with output bit-stream data was performed by using the Audio Precision APx525. The inductance, capacitance and resistance (LCR) meter was used to measure the chip capacitor to compare with the measurement results by the proposed prototype IC for measurement evaluation. The temperature chamber was used to evaluate the performance of the on-chip temperature sensor.

### 3.3. Measurement Results

**Figure 11:**
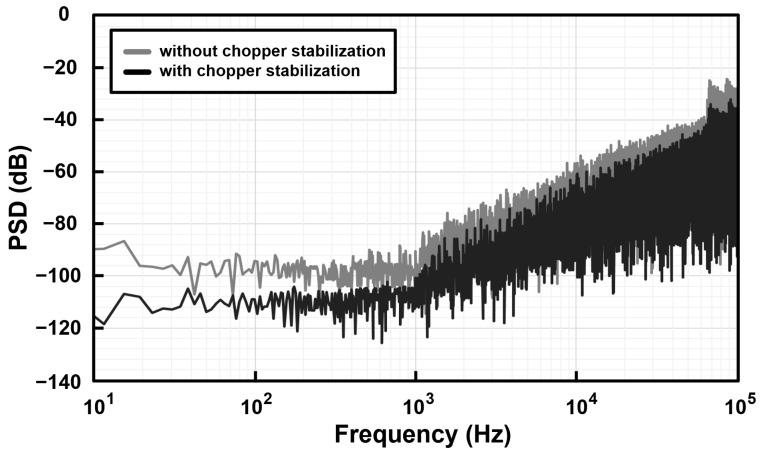
FFT measurement results of the proposed reconfigurable sensor analog front-end IC.

The FFT measurement results of the driving mode of the capacitive microsensors in the proposed reconfigurable sensor analog front-end IC are shown in Figure 11. The FFT was achieved by the bit-stream data FFT length of 65,536 points with a Blackman–Harris 3-term window. The gray line shows the measurement results without the application of the chopper stabilization technique and the black line shows the measurement results with the application of the chopper stabilization technique. The FFT result shows enhanced reduction of noise by the chopper stabilization technique implemented in the proposed scheme.

**Figure 12:**
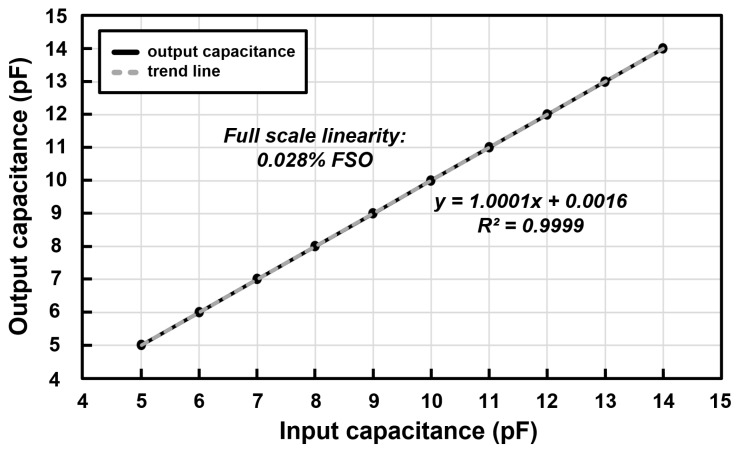
Linearity simulation results of the proposed reconfigurable sensor analog front-end.

**Figure 13:**
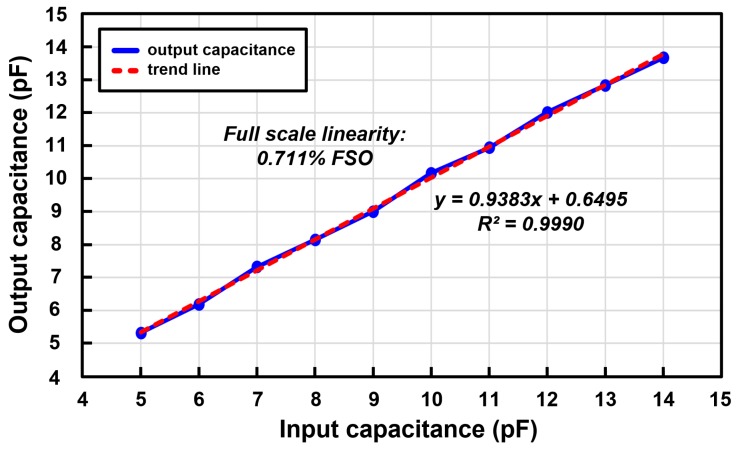
Linearity measurement results of the proposed reconfigurable sensor analog front-end.

The simulation result of the capacitance linearity is shown in Figure 12. The output capacitance is indicated by the black line and the gray line indicates the trend line of the simulated output capacitance. The simulated nonlinearity is 0.028% FSO. The simulation was proceeded by Cadence Virtuoso for comparison with the measurement result. The capacitance linearity measurement result of the driving mode of the capacitive microsensors in the proposed scheme is shown in Figure 13. The capacitance linearity was measured by changing the input capacitance and acquiring the output digital codes. The output capacitance is indicated by the blue line and the red line indicates the trend line of the measured output capacitance. The measured nonlinearity is 0.711% FSO. The input capacitance was connected and measured using the LCR meter, and then connected to the analog front-end circuit; thus, the measurement results include the relatively large non-linearity because of the parasitic capacitance from the measurement environment.

**Figure 14:**
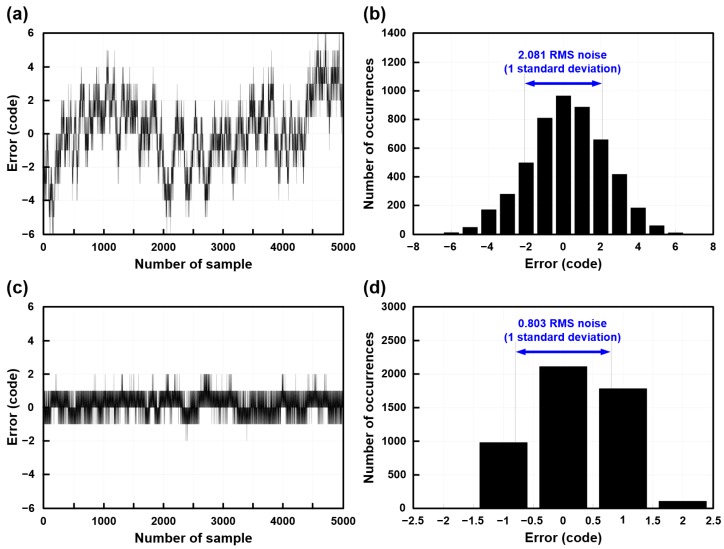
Measured output codes of the proposed reconfigurable sensor analog front-end IC. (**a**) Output code error without chopper stabilization; (**b**) Histogram of output code error without chopper stabilization; (**c**) Output code error with chopper stabilization; (**d**) Histogram of output code error with chopper stabilization.

The measured output codes of the capacitive microsensors of the proposed scheme are shown in Figure 14a–d. The output code error without chopper stabilization is shown in Figure 14a and its histogram is shown in Figure 14b. The output code error with the application of the chopper stabilization is shown in Figure 14c and its histogram is shown in Figure 14d. Five thousand sets of data values with fixed input capacitance of 6 pF were acquired. Figure 14a shows the peak-to-peak (*P*-*P*) noise of ±6 code variation without the application of the chopper stabilization. Figure 14c shows the measurement results of the code variation with the application of the chopper stabilization, which improved the code variation to ±2. The root mean square (*RMS)* noise is improved from 2.081 to 0.803, as shown in Figure 14b and Figure 14d. The measured input referred capacitance *RMS* noise is 0.180 fF without the application of the chopper stabilization and 0.069 fF with the application of the chopper stabilization. The *RMS* noise and *P*-*P* noise can be acquired by the standard deviation and difference of the maximum and minimum results of the data. The effective resolution and *P*-*P resolution* can be acquired for performance evaluation. The effective resolution can be calculated as Equation (17) and the *P*-*P resolution* can be calculated as Equation (18) from the histogram results [29]:

(17) $$Effective\;resolution=\log_{2}(\frac{P-P\;Range\;(LSBs)}{RMS\;Noise\;(LSBs)})$$

(18) $$P-P\;resolution=\log_{2}(\frac{P-P\;Range\;(LSBs)}{P-P\;Noise\;(LSBs)})$$

The measured effective resolution and *P*-*P resolution* of the proposed scheme without the application of the chopper stabilization are 14.9-bit and 12.4-bit, respectively. The measured effective resolution and *P*-*P resolution* with the application of the chopper stabilization are 16.3-bit and 14-bit, respectively.

**Figure 15:**
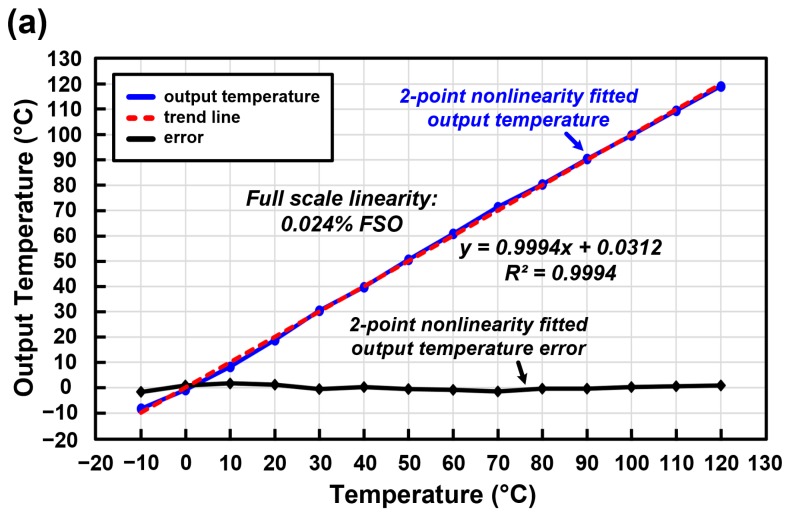

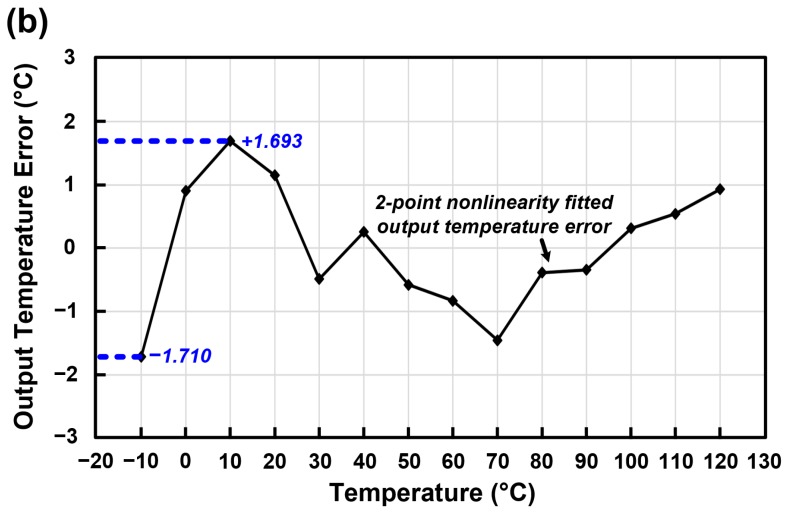
Measured output data of the temperature sensor of the proposed sensor analog front-end. (**a**) Measured output temperature data; (**b**) Error of the two-point nonlinearity fitted output temperature.

The measured output data of the temperature sensor with the proposed reconfigurable sensor analog front-end IC are shown in Figure 15a and Figure 15b. The measured temperature range is −10 °C to 120 °C. Figure 15a shows the measured output temperature data. The blue line represents the output temperature with two-point nonlinearity fitting. The red line is the trend line of the measured output temperature and the black line represents the error of the two-point nonlinearity fitted output temperature, which is shown in detail in Figure 15b. The measured nonlinearity is 0.024% FSO and the output temperature error is −1.710/+1.693 °C.

## 4. Discussion and Conclusions

The performance summary and comparison of previous works with the proposed reconfigurable sensor analog front-end scheme is shown in Table 1. The figure-of-merit (*FoM*) for the performance comparison can be calculated as given in Equation (19) [30]:

(19) $$FoM=\frac{P_{total}\cdot T_{measurement}}{2^{resolution}}$$

The performance summary and comparisons show that the proposed work achieves high effective resolution, low power consumption, and low *FoM*. The power consumption of 0.843 mW and *FoM* of 13.06 pJ/step show improvements compared with previous works with similar architecture of discrete time delta-sigma CDC. The wide range of input capacitance with 16.7 pF of the proposed CDC is also an advantage compared to previous works, which makes it suitable for various capacitive microsensor applications.

This paper presented a reconfigurable sensor analog front-end using low-noise chopper-stabilized delta-sigma CDC. The proposed scheme can drive both capacitive microsensors and voltage signals by direct conversion. A fully integrated temperature sensor is implemented in the proposed reconfigurable sensor analog front-end for various applications. A low-noise technique with chopper stabilization was implemented to achieve a low-noise characteristic. The prototype IC was fabricated as per the standard 0.18-μm 1P6M CMOS process. The proposed scheme occupies a total active area of 5.37 mm^2^ and the total power consumption is 0.843 mW with a 1.8 V power supply. It achieves an effective resolution of 16.3-bit and measured input referred capacitance RMS noise of 0.069 fF. The measured capacitance nonlinearity and the measured temperature nonlinearity are 0.711 % FSO and 0.024% FSO, respectively. The proposed sensor analog front-end can be flexibly adopted in various IoT sensor system applications with reasonable performance due to its reconfigurable scheme.

## Figures and Tables

**Table 1:** Performance summary and comparisons.

Specification	This Work	[20]	[31]	[32]	[33]	[34]
Modulator order	DT 2nd	DT 1st	DT 2nd	DT 2nd	DT 4th	DT 2nd
Process (μm)	0.18	0.35	0.35	-	0.25	0.18
Re-configurability	Y	N	N	N	N	N
Supply voltage (V)	1.8 (core)/3.3 (pads)	3.3	3.0	5.0	2.5	2.6
Power consumption (mW)	0.843	1.44	60	3.75	6	2.34
Measurement time (ms)	1.25	0.128	1000	20	500	3.07
Effective Resolution (bit)	16.3	11.0	20.0	19.4	20.3	17.4
Capacitance range (pF)	16.7	1	1	8	5.3	10
*FoM* (pJ/step)	13.06	90	57220	108	2300	37
Active area (mm^2^)	5.37	0.05	0.65	-	2	0.67
